# Student Health Ambassadors: A New Program to Promote Health among the Adolescent

**DOI:** 10.30476/ijcbnm.2020.46761

**Published:** 2021-01

**Authors:** Naeimeh Sarkhani, Shahzad Pashaeypoor, Reza Negarandeh

**Affiliations:** 1 Nursing and Midwifery Care Research Center, School of Nursing and Midwifery, Tehran University of Medical Sciences, Tehran, Iran; 2 Department of Community Health and Geriatric Nursing, School of Nursing and Midwifery, Tehran University of Medical Sciences, Tehran, Iran

**DEAR EDITOR**

Health promotion is the process of enabling people to increase control over their health and its determinants, and thereby improve their health. Through the Ottawa Charter, World Health Organization (WHO) defined health promotion as “the process of empowering individuals to increase their control and improve their health”. ^1^ WHO and the United Nations Educational, Scientific and Cultural Organization (UNESCO) have launched a new initiative called “Making Every School a Health Promotion School” through the development and promotion of global standards for health promotion in schools. The initiative will help more than 2.3 billion students and the WHO to achieve the motto of “one billion healthy lives” by the end of 2023. ^2^

Today, 1.2 billion adolescents in the world make up more than one-sixth of the world’s population; also, more than 80% of them are teenagers and spend one-third of their time in school. ^2^ Highlighting the issue of health for this group, especially the adolescents, is important because their physical, mental, and social health guarantees the current and future health status of the society. Therefore, the purpose of school health is to provide, maintain, and improve the physical and mental health of adolescents and ultimately communities. Adolescents need to know how to take care of themselves and what measures to take for their health. ^3^

Given that the WHO aims to promote health among children and adolescents, special emphasis should be placed on the implementation of health promotion programs. Teaching based on the appropriate approaches to promote health and to care for adolescents will improve the health of the community. In addition, it can help reduce the financial costs of chronic diseases and their growing prevalence. ^4^

For this purpose, the Health and Welfare Office of the Ministry of Education in Iran in cooperation with the Education and Health Promotion Office of the Ministry of Health and Medical Education (MOHME) has signed an executive agreement in the form of a program for student health ambassadors. This program was designed to provide grounds for mobilizing students and to provide health ambassadors with a plan to make efforts to comprehensively implement biological and physical training. The program is based on the peer education approach, which is a coherent program designed to build an effective peer network to encourage students to improve their health. Accordingly, 10% of the diligent, interested, and volunteer students in the school and also at least one qualified student from each class will be selected by the healthcare provider or the coordinator. They will be provided with a self-care booklet and a healthy lifestyle. Then, the healthcare providers in each school are asked to provide the necessary information to the health ambassadors through holding special training sessions (group discussion, workshop, panel, and role play). Consequently, they should transfer health information to their peers at school. Student Health Ambassadors are referred to those volunteer students who are interested in group activities in various fields of health (physical, psychological, social, and spiritual). They should be able to play a pivotal and effective role in promoting health and self-care not only in schools, but also among their families. This is a newly established program that was endorsed in 2016-17. The “Health Ambassadors” program was developed for the elementary and secondary schools in urban and rural areas in cooperation with the Office of Health for Population, Family and School and Education and Health Promotion Office in the MOHME. ^5^

This program is also faced with many challenges, indeed. Given the need for an educator and the establishment of an intimate and friendly relationship between the educator and the audience as the main principles of health education, it seems that the presence of a health educator or a school nurse is one of the priorities and underlying foundations of the project. Therefore, the appropriate physical and human context for health education and promotion should be provided to be used efficiently. Despite the measures taken, the “Health Ambassadors” program still seems to be in the pilot stage; however, it has been welcomed by many schools and students as a health ambassador. Developing a sense of responsibility for one’s health as well as the health of others is one of the main objectives of this program. Therefore, after evaluating and resolving its challengesas a start-up project, the “Health Ambassadors” project can have significant short-term and long-term consequences.

Therefore, developing a plan such as the “Student Health Ambassadors Project” is a dynamic way of thinking in pursuit of sustainable development in society. It is noteworthy that such a proposal can be implemented in other countries as well. However, it seems that highlighting the presence of an expert in health education and promotion is one of the most important issues that should be taken into account by the executives in order to improve the implementation of the Health Ambassadors Project. Community health nurses with sufficient knowledge about the developmental stages of adolescents, principles of school health, nursing procedure, communication process, and educational methods can provide adolescents with the necessary health education in schools. Accordingly, we recommend employing community health nurses in schools to achieve more desirable results in terms of the implementation of school health programs including the “Health Ambassadors Project” in the future. Consequently, this will lead to better health education as well as the promotion of healthy behaviors. 
